# Ultrasonication Influence on the Morphological Characteristics of Graphene Nanoplatelet Nanocomposites and Their Electrical and Electromagnetic Interference Shielding Behavior

**DOI:** 10.3390/polym16081068

**Published:** 2024-04-11

**Authors:** Ignacio Collado, Alberto Jiménez-Suárez, Antonio Vázquez-López, Gilberto del Rosario, Silvia G. Prolongo

**Affiliations:** 1 Materials Science and Engineering Area, Escuela Superior de Ciencias Experimentales y Tecnología, University Rey Juan Carlos, Tulipán Street, 28933 Móstoles, Madrid, Spain; alberto.jimenez.suarez@urjc.es (A.J.-S.); silvia.gonzalez@urjc.es (S.G.P.); 2 Technological Support Center, University Rey Juan Carlos, Tulipán Street, 28933 Móstoles, Madrid, Spain; gilberto.delrosario@urjc.es; 3 Instituto de Tecnologías para la Sostenibilidad, Universidad Rey Juan Carlos, Tulipán Street, 28933 Móstoles, Madrid, Spain

**Keywords:** epoxy, GNPs, shielding, percolation

## Abstract

Graphene nanoplatelets (GNPs)/epoxy composites have been fabricated via gravity molding. The electrical and thermal properties of the composites have been studied with variable GNP type (C300, C500, and C750, whose surface areas are ~300, 500, and 750 m^2^/g, respectively), GNP loading (5, 10, 12, and 15 wt.%), and dispersion time via ultrasonication (0, 30, 60, and 120 min). By increasing the time of sonication of the GNP into the epoxy matrix, the electrical conductivity decreases, which is an effect of GNP fragmentation. The best results were observed with 10–12% loading and a higher surface area (C750), as they provide higher electrical conductivity, thereby preserving thermal conductivity. The influence of sonication over electrical conductivity was further analyzed via the study of the composite morphology by means of Raman spectroscopy and X-ray diffraction (XRD), providing information about the aspect ratio of GNPs. Moreover, electromagnetic shielding (EMI) has been studied up to 4 GHz. Composites with C750 and 120 min ultrasonication show the best performance in EMI shielding, influenced by their higher electrical conductivity.

## 1. Introduction

Polymer nanocomposites reinforced with carbonaceous nanomaterials comprise an evolving matter of study due to their unique mechanical, electrical, and thermal properties [1]. Among the carbonaceous fillers, graphene stands out, which is attributed to its intrinsic unique properties. These properties arise from its sp^2^ hybridized carbon–carbon network, forming a 2D planar structure, with a high surface area and high aspect ratio (length/width). Commonly, graphene is manufactured and employed in the form of submicron aggregates called graphene nanoplatelets (GNPs). Notably, epoxy (EP) thermosets are one of the main resins that can benefit from the use of graphene as a reinforcement [2]. Their synergistic performance leads to improved dielectric properties [3], corrosion prevention [4], or enhanced fracture toughness [5], among other remarkable features. Epoxy-reinforced graphene nanoplatelets have found application in fields such as the aerospace industry, mostly as adhesives [6], anti-icing and deicing coatings [7], or as electromagnetic interference shielding (EMI) devices [8].

For the latter application, epoxy/graphene nanocomposites are valuable candidates, as they possess higher electronic conductivity and proper thermal management, leading to protection against electromagnetic radiation and generated heat during performance. This feature highlights their potential for protection against electromagnetic radiation for new microelectronics and communication devices. In fact, several nanomaterials have been employed as reinforcement for EMI shielding in the EP matrix. To mention a few, we can highlight carbon nanotubes (CNTs), graphene oxide (GO), and carbon black [9,10]. Among these, graphene nanoplatelets (GNPs) have raised recent interest due to their less expensive price and largescale production [11], as well as their high EMI shielding performance [9,10,12]. In fact, their combination with other fillers offers extremely high shielding performance [13].

However, several key challenges need to be studied to optimize the electrical conductivity and EMI shielding performance of GNP/EP composites. Firstly, GNP/epoxy composites are nonconductive for low filler contents until they reach a critical concentration value (percolation threshold) [14] for which the formation of conductive pathways allows a rapid increase in electrical conductivity. This filler content can range from 4 to 6 wt.% [15] in the uncured epoxy mixture and increase up to 7–10% for cured epoxy [11,16].

Secondly, a proper dispersion of GNPs in the EP matrix is crucial to achieve the desired properties and allow for the formation of the conductive pathways. The manufacturing imposes a strong impact that affects the quality of the GNPs [17]. Some studies have highlighted the importance of several parameters (time, temperature) and their influence on the thermal or electrical behavior of GNP/epoxy nanocomposites [18,19,20]. The dispersion quality can be also strongly influenced by the morphology [21,22] of the embedded material; therefore, the surface area of GNPs might play a crucial role. Also, a higher surface area might be related to an increased presence of defects in GNPs [23].

Lastly, assisted-tip sonication GNP/EP dispersion can produce undesired effects [24], enhancing significant degradation of the graphene structure, with low exfoliation times being preferable to prevent GNP fragmentation. The time of tip ultrasonic operation is considered to affect not only the exfoliation of the GNP flakes but also the lateral size of GNPs [25]. GNPs’ electrical properties are also directly linked to the state, damage, and number of platelets. Thus, it is fundamental to control sonication power and time to avoid any damage to the GNPs that will detriment the electrical conductivity, as high power can produce oxidation or graphitization of the GNPs [24]. Conclusively, the relation of the aforementioned parameters, which are closely related, involves a fundamental study for the optimization of GNP/epoxy performance.

Herein, we report the manufacturing of nanocomposites based on reinforced epoxy resin with GNPs (GNP/EP) and their thermal and electrical properties, with variable loading and different graphene nanoplatelet surface areas (commercially available as C300, C500, and C700, whose surface areas are ~300, 500, and 750 m^2^/g, respectively) and electromagnetic shielding performance. Variable loading, alongside proper sonication times, leads to changes in the exfoliation process of GNPs, directly affecting the thermal and electrical conductivity. The electrical conductivity is analyzed as a function of the GNP properties (aspect ratio and lateral size) and the dispersion quality. Finally, the EMI shielding performance is studied in the GHz range.

## 2. Materials and Methods

### 2.1. Materials

GNPs’ (graphene sheets) morphology is constituted by small stacks of platelet shape. Three kinds of grade C GNPs (C300, C500, and C750, XG Science, Lansing, MI, USA) were employed. The manufacturer estimates that the average surface areas are ~300, 500, and 750 m^2^/g for C300, C500, and C750, respectively. It is worth mentioning that grade C consists mostly of particles with sizes under the micra. The particle diameter is often under 2 μm. Further information regarding the GNP powders can be found in Table 1.

The polymer matrix was an epoxy resin (Araldite LY556, Huntsman, Tienen, Belgium) with a formulation based on bisphenol A diglicidyl ether (DGEBA) cured with an aromatic polyamine-based hardener (araldite XB3473, Huntsman, Tienen, Belgium), based on a mixture of diethyltoluenediamine (87–93%) and 1,2-diaminocyclohexane (7–13%). Table 2 summarizes the significant characteristics of the two components. It is worth mentioning that the viscosity of the final mixture at 23 °C is 5600 (mPa s) and 800 (mPa s) at 40 °C.

### 2.2. GNP/Epoxy Processing and Sample Preparations

**Figure 1:**
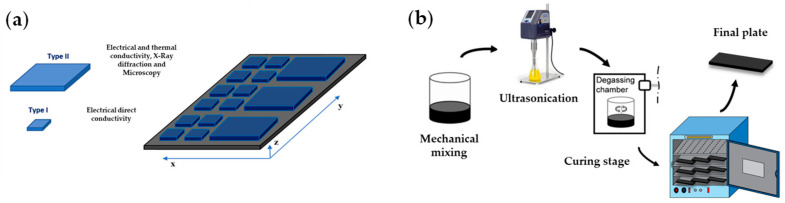
Graphical representation of the samples for different experimental tests employed (**a**). Type I (10 × 10 × 2 mm^3^) and Type II (40 × 40 × 2.5 mm^3^). Process flow diagram for the manufacturing systems (**b**).

The manufacturing process is reported elsewhere [8], differing from this work in the curing cycle employed. In summary, xGnP at concentrations of 5, 10, 12, and 15 wt.% was mixed with neat epoxy monomer and dispersed via an ultrasonication probe for variable times (30, 60, and 120 min). Then, the sample was degassed in vacuum conditions and magnetic stirring at 80 °C for 15 min, and the hardener was added in a mix ratio of 23:100 parts by weight. The samples were finally cured at 6 h 140 °C and 2 h at 160 °C in an oven (the cure cycle was determined from the manufacturer’s specification sheet, and it was ensured using differential scanning calorimetry (DSC) that the cure is complete), leaving a Tg of 180 ± 3 °C without GNP. Various geometries were prepared for the different characterization techniques, aided with a CNC milling machine from 286 cm^3^ plates, as observed in Figure 1.

To obtain a homogeneous dispersion, a sonicator probe was employed (UP400S, Hielscher Ultrasound Technology Teltow, Germany), with a power of 400 W and a frequency of 24 kHz and configured with a cycle time of 0.5 or 50% and a low amplitude of 50%. As for the applicator, a 22 mm diameter (Sonotrode H22) was used to infer uniformly throughout the sample. It is important to note that the sonication was performed at a maximum temperature of 95 °C and was carried out on a 100 cm^3^ batch (to produce a higher quantity than classically observed in these types of nanocomposites, which is far from the industrial reality), placing the tip of the sonotrode at mid-height. The vessel used was 250 mL to optimize the sonication efficiency.

### 2.3. Composite Characterization

#### 2.3.1. Electrical Conductivity

The direct current (DC) electrical conductivity was obtained in the xy plane using the 4-wire connections method to minimize or eliminate the effects of cable resistance. Type II specimens (Figure 1) in agreement with ASTM D257. A Source Meter Keithley 2410 was employed, while the voltage applied was in a variable range (0–1000 V), and the recorded current was limited with a compliance of 1 A (up to 25 V) or 0.02 A (from 25 V to 1000 V). KEITHLEY 6514 (Cleveland, OH, USA) was used as an ammeter to measure the pristine and 5 wt.% samples.

The electrical resistance, R, was obtained using the intensity–voltage (I–V) slope, applying voltages in the 0–1000 V range. Then, the electrical conductivity was calculated using Equation (1), where “t” is the distance between the electrodes (average thickness of the specimen), “A” is the effective area of the measuring electrode for the particular arrangement employed, and “Gv” is the conductance as follows:

σ = (t/A)Gv
(1)

Specifically, voltages were used to obtain a linear I–V relationship, facilitating the calculation of the resistance by using ohm’s law (V = I × R). Measurements were made on the specimens in the direction parallel to the xy plane.

#### 2.3.2. Thermal Conductivity

Thermal conductivity measurements were obtained with Fox 50 instrument using Win-Therm 50V3 software (TA Instruments, 159 Lukens Drive, New Castle, DE, USA) with the type II samples in the Z direction. The thermal properties of the composite as a function of temperature were conducted with a resolution of 0.1 W/mK.

To carry out the tests, a temperature of 40 °C was set, and the type II specimens were cut in circular geometry and roughed in a polishing machine until a uniform thickness was achieved. The calculation of thermal conductivity is based on the heat transfer equilibrium between the two cylindrical plates. The results were obtained and analyzed using Win-Therm 50V3 software. For the calculation of thermal conductivity, the expression (2) is used as follows:

(2) $$K=\alpha \cdot C_{p}\cdot \rho$$

where α is the thermal diffusivity, $C_{p}$ is the specific heat, and ρ is the density of the material.

#### 2.3.3. Scanning Electron Microscopy (SEM), Transmission Electron Microscopy (TEM) and Optical microscopy

For the preparation of the different specimens, they were pressed into the mold and filled with EpoFix Resin (Struers S.A.S., Champigny-sur-Marne, France). Subsequently, the already drawn sample was polished using 600, 1200, 2500, and 4000 μm sandpaper and, finally, a 0.05 μm alumina solution. A sputtering or bath treatment was applied (Emitech K550X model Quorum Technologies, Lewes, UK). Gold was used as a conductive element, preventing the SEM electrons from accumulating on the surface of the sample. The process is performed under a vacuum and while applying argon gas to achieve an inert atmosphere. The microscope used was an SEM S-3400N model (Hitachi, Tokyo, Japan). The photographs were taken with an acceleration potential of 15,000 V and a current intensity between 100,000 and 125,000 nA.

To determine the GNP exfoliation, the samples were prepared via cryofracture with the use of liquid nitrogen. The cross-section was coated with a 2 nm-thick platinum layer by sputtering. The images were obtained with a high-resolution Nova NanoSEM 230 (FEI Company, Hillsboro, OR, USA).) under a high-vacuum regimen. To analyze the images, a free software was employed—FIJI (ImageJ 1.53c, NIH).

The morphological study of pristine graphene nanoparticles was carried out using transmission electron microscopy (TEM, Phillips Tecnai F20, 200 kV, FEI Company, Hillsboro, OR, USA). The powdered samples are dispersed in acetone in an ultrasonic bath, and a drop is deposited on a copper grid to facilitate the study of individual particles when a thickness measurement is possible, as well as the visualization of atomic planes (002). For the observation of the nanocomposite, cryogenic ultramicrotomy cutting is performed in a Leica EM FCS equipped with a diamond blade so that the thickness of the cuts is sufficiently small, of the order of 40 nm, to allow study using this technique. For sample preparation without sonication, graphene was manually dispersed in N-methylpyrrolidone (NMP) and deposited on a membrane.

Optical images were obtained using light-transmitted optical microscopy (TOM) before the hardener addition (prior to the curing stage) with a Leica DMR microscope (Leica Microsystems, Wetzlar, Germany) equipped with a camera—the Nikon Coolpix 990 (Nikon, Tokyo, Japan). The possible variation in the agglomerate size of the graphene particles is also studied because, being a micrometric material, they are observable using this technique. Digital image analysis, using FIJI (ImageJ 1.53c, NIH), was performed for determining the agglomerate size.

#### 2.3.4. X-ray Diffraction (XRD)

Diffractograms were obtained using X-ray radiation (CuKα, 1.5406 A) in a Panalytical’s X’Pert PRO working in a 2θ scanning configuration at 45 kV and 40 mA. The angle range was between 5 and 90°. The program used in the treatment and identification of the diffractograms was X’Pert High Score Plus (version 3.0, Malvern Panalytical, Enigma Business Park, Grovewood Road, Malvern, UK). X’pert HighScore Plus software 4.0 was employed to analyze the data. X-ray diffraction was performed on uncured dispersed samples, as well as the TOM.

The diffraction spectra of the nanoparticles and the resulting nanocomposite materials are analyzed using the X’Pert HighScore Plus program to obtain the values corresponding to the full width at half height (FWHM), peak position, and intensity to calculate the size of the crystalline domain in each of the cases. The calculation of the particle size is carried out using the Scherrer equation [28,29], assuming no deformation of the glass, as follows:

(3) $$L_{hkl}=\frac{k\cdot \lambda }{\beta_{0}\cdot \cos\theta }$$

where *L* is the size of the crystal in the direction perpendicular to the plane (*hkl*), λ is the wavelength of the radiation source, $\beta_{0}$ is the FWHM, and θ is the angle at which diffraction occurs.

#### 2.3.5. Raman Spectroscopy

All micro-Raman measurements were taken using HORIBA Jobin Yvon Lab RAM HR 800 (HORIBA Europe GmbH, Lier, Belgium) with an excitation wavelength of *λ* = 514.6 nm (2.41 eV) Ar ion laser in a backscattering configuration. A 100× objective configuration, 200 μm hole, and the laser in high-density mode were used. The total acquisition time was 30 s, accumulating 5 measures, and the laser power did not exceed 5 mW to avoid inducing damage. At least three measures for each sonication condition over 10 and 12 wt.% were carried out, displaying the average signal.

The intensity, full width half maximum (FWHM), and peak positions data of D, G, and 2D positions were obtained by deconvoluting the data of all Raman spectra using single Lorentz functions. This fitting was performed with the software Origin Pro v.9 via the automatic parameter initialization. For normalization and the conditioning signal, SpectraGryph software (https://www.effemm2.de/spectragryph/, accessed on 7 April 2024) was used.

#### 2.3.6. Electromagnetic Shielding Measurements

The near-field electromagnetic shielding effectiveness method using a vector network analyzer (N9923A FieldFox, Keysight, Santa Rosa, CA, USA) was utilized to measure electromagnetic interference (EMI) shielding effectiveness (SE) in the frequency range of 1.5–6.0 GHz of the bulk type II materials (Figure 1). Three specimens were tested for each component (the result shown is the average of the signals). The results obtained for the near electric field (SE_E_) and near magnetic field (SE_M_) shielding are presented, together with the justification of the interest of the obtained values and their possible origin.

All measurements were made using the NA mode, by means of which the S-parameters relating the signals emitted (in) to those received (out) can be obtained. Full 2-port calibration was performed, and N-type connectors were used between the VNA ports and the near-field probes. Subsequently, the *S*_21_ parameter was evaluated by measuring the energy radiated by the port one probe, with the measurement being repeated with the absorber structure placed between both probes [30].

The EMI-SE shielding effectiveness is defined in decibel form, where |*S*_21_| is the ratio between the power transmitted through the material to the incident power (forward transmission coefficient) [31], as follows:

(4) $${\mathrm{SE}}_{x}(\mathrm{Total})=10\cdot \log\frac{1}{{\left|S_{21}\right|}^{2}}$$

where x can be either referring to e (electric field) or m (magnetic field). In this system, it is not possible to separate shielding into reflection and absorption contributions because multiple reflections cannot be neglected (the set < 10 dB, generating that the thickness of the shielding material is not greater than the skin depth).

## 3. Results and Discussion

### 3.1. Characterization of GNP

Figure S1 shows TEM images of the evolution of a nanoplatelet with sonication time. It can be observed that the C300 nanoparticle experiences a shortening of the lateral size with sonication time, and it is also clear that these nanoplatelets are composed of several stacked mono- and bilayer graphenes, thus exfoliating over time. In addition, TEM shows that the damage is generated because these composite nanoplatelets separate and new edges are formed. These new edges also induce the formation of folds because they act as zones for the release of the mechanical energy absorbed during sonication. All the effects induced during sonication, with the exception of exfoliation, which can also be observed in Figure S1, could contribute to the decrease in electrical and thermal conductivity.

To identify the real thickness of the nanoparticles, a morphological analysis is performed using scanning and transmission electron microscopy. Figure S2 shows the measurement of a nanoparticle that represents the average for C300 pristine, coinciding with the thickness measurement obtained using XRD. Figure S3 shows the images captured using STEM for the quantification of the lateral size of the graphene. The result of the analysis shows lateral sizes of 1.12 ± 0.14; 0.58 ± 0.08, and 0.29 ± 0.04 µm for graphene C300, C500, and C750, respectively. This result is very similar to that indicated by the manufacturer and to that observed in other articles.

The elemental quantification results of the GNP/epoxy composites were obtained via energy-dispersion spectroscopy (EDS). With the limitation of the technique for light elements, the element analysis is presented in Table S1. In general, GNPs are considered purer regarding the content of elemental carbon [32]; however, from the results, it is clearly observed that upon surface area increase, the oxygen content is higher. This is because a larger surface area increases the probability of the appearance of surface defects or functionalization caused by the need to minimize the energy of the GNP, which in turn, allows graphene with a larger surface area (C750) to present a smaller thickness because the oxygen functional group and the wrinkles or folds prevent the restacking of the graphene sheets. The origin of the oxygen (energy minimization) can be seen from two points of view. Graphene, being a two-dimensional material, has a large surface area. This means that it is exposed to a large amount of oxygen molecules in the air, which adsorb to its surface. On the other hand, we would have the influence of defects in the graphene structure on adsorption, as the edges or irregularities (abundant in C750) act as adsorption sites for oxygen molecules.

### 3.2. Electrical Conductivity

EMI shielding is a parameter dependent on high electrical conductivity for incident wave reflection, as it has a significant impact on its dielectric properties. Thus, tuning the conductivity is a key mechanism to achieve high electromagnetic shielding [33]. It has been widely discussed that GNPs grant high electrical conductivity when their concentration within the polymer host matrix exceeds a critical concentration (called percolation threshold), often around values between 7 and 8 wt.% [11,16]. As a first step in this work, GNP/epoxy samples were prepared with variable ultrasonication time (30, 60, and 120 min) in different concentrations (0, 5, 10, 12, and 15%), and a 20% value is extrapolated (based on information contained in bibliography [26,34]) due to the difficulty of manufacturing it properly.

**Figure 2:**
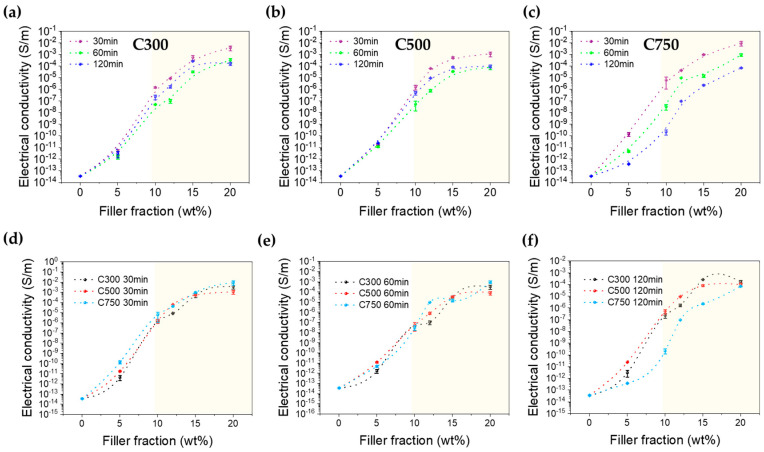
Electrical conductivity of GNP/epoxy nanocomposites as a function of GNP content for the different graphene types (**a**) C300, (**b**) C500, and (**c**) C750. Yellow region separates the percolation region. The electrical conductivity as a function of the sonication time for each graphene type is shown in (**d**) for 30, (**e**) 60, and (**f**) 120 min.

As observed in Figure 2, when the GNP content was below 5 wt.%, the electrical conductivity of the nanocomposites was higher than that of pristine DGEBA but insufficient to reach a highly conductivity value, most likely due to the GNPs dispersed into the epoxy matrix not forming a conductive network [11]. Higher GNP content, such as 10%, increases conductivity, up to 10^−5^ S/m, from insulating to semiconductor-like behavior.

The nanocomposites obtained after fabrication present a different percolation threshold from that classically obtained with graphene and carbon nanotubes (Figure S4), as they present a much lower slope in part 2 (the one above the percolation threshold). This makes it difficult to fix a value for the percolation threshold, although it is clear that the value is higher for C750, then for C500, and finally for C300. The origin of this behavior is that the lower density of C750 implies that for the same mass, it occupies a larger volume, which means that the particles are closer to each other. In addition, by having a larger surface area, it also reduces the distance between GNPs by allowing part of the volume (roughness and porosity) of the GNP to be occupied by the polymer. It is well known that filler geometry affects electrical conductivity [21]. A higher surface area creates more through-thickness and in-plane conductive networks and pathways for charge movement [31], which increases electrical conductivity. On the other hand, it is true that a larger lateral size allows for a greater probability of connection at certain points, which forms percolated pathways and increases properties such as electrical conductivity. Then, if we study it by zones (zones above and below 10% by weight), we can observe in Figure 2d–f the following:

- For the content zone below 10% by weight, the higher surface area and lower density of GNP C750 results in a higher amount of nanoparticles and, consequently, a greater chance of forming more conductive networks and decreasing the distance between adjacent nanoparticles. This has also been observed with other polymer matrices by other authors [27,34].
- For contents above 10%, the inherent conductivity of the nanofiller determines the electrical conductivity of the composite, and the size and shape of the filler have little effect on the electrical conductivity of the composite, so that the difference between the conductivities depending on the type of GNP decreases. This is possible because the number of contacts increases to such an extent that the contact resistance dominates over the tunnel resistance, and the influence of the interparticle distance is lost.

Comparing graphs (a), (b), and (c) in Figure 2, we can conclude that sonication time affects graphene C750 the most, generating a continuous and steeper drop in conductivity with increasing sonication time. If we look at graphs (d), (e), and (f), which compare as a function of sonication time each graphene type, we find that below the percolation threshold, for 30 min, C750 shows the highest conductivity, while for 60 and 120 min, C500 dominates. Above the percolation threshold, the differences are minimized, and only at 120 min it is observed that C750 shows a much lower conductivity regardless of the weight percentage of GNP.

The origin of the loss of electrical conductivity decrease over time is because the sonication process achieves nanofiller disaggregation by means of cavitation forces, which both led to the exfoliation of the GNPs and the breakage for nanoparticles. However, those same jets have a high probability of causing damage and generating defects in the graphitic structures, such as the creation of folds and wrinkles [22], which lower the conductivity [35].

Moreover, ultrasonication time has been attributed as a clear cause of inducing defects and oxygen. Per the electrical results and with the focus of high EMI shielding, focus was limited to those samples above the percolation threshold.

At C300 and C500, a drop in conductivity is observed as the sonication time increases, significantly between 30 and 60 min. This drop in conductivity can be explained by the amorphization transformation [36] of the graphene structure. However, between 60 and 120 min, a rise in conductivity is exhibited because the appearance of defects and amorphization have stabilized [24], while exfoliation favors the rise in conductivity (Table S5), thus giving rise to an increase in exfoliation without damage [24]. This, together with a slight drop in lateral size after 60 min (Table S3), implies that the aspect ratio hardly changes (for a single GNP), which confirms the hypothesis showing that the only influential parameter would be dispersion, which improves with sonication time from 60 to 120 min (Figure S10), leading to the increase in electrical conductivity.

In addition, other authors have shown that there is a sonication time for which the size of the carbon nanostructures does not decrease [37], varying this stabilization time between 1 and 3 h due to the existence of a dependence on the sonication energy used, the type of GNP used, and the percentage by weight of the GNP used. At this point, it is important to understand why this phenomenon occurs for C300 and C500, but not for C750. In C750, this stabilization time has not been reached after 2 h of sonication, and it is observed by Raman that the damage continues to increase after 60 min (Table S7), and that fragmentation is still present (Table S3), unlike what occurs in C300 and C500. The origin of this difference in behavior against sonication must lie in its major differential characteristic, which is the aspect ratio, being between two and four times higher in C750. A higher aspect ratio implies a higher ratio between lateral size and thickness, facilitating the breakage of the GNP.

For the sonication conditions and materials used, the damage generated and the exfoliation can be taken as linear combinations of lateral size, surface area, and thickness. Lateral size and specific area are directly proportional to the damage generated, and inversely proportional to the exfoliation, because based on classical mechanics, the increase in lateral size implies a greater probability of fragmentation of the GnP due to the generation of greater moments of force, and the increase in surface area implies the existence of a greater roughness in the GnP, which leads to a greater number of points that can act as stress concentrators. On the other hand, the thickness is inversely proportional to the damage generated because a lower thickness implies a lower resistance to the deformation caused by the jets during sonication.

A logical justification based on probability, geometry, and mechanics would be to affirm that a greater specific area, maintaining constant dimensions, leads to a probabilistic increase in the existence of greater rupture events, which from the mechanical point of view, are favored by the possibility of the appearance of greater net stresses linked to larger dimensions, such as bending moments. Increasing the dimensions, for a constant geometry, would lead to the same; however, increasing the thickness (number of graphene sheets in the GNP) would lead to an increase in the resistance to damage. In our case study, the larger the specific area, the smaller the thickness and the larger the lateral size.

The lateral size decreases similarly for all three GNP types after 60 min; however, the number of lamellae is much smaller for GNP C750, which also has a larger specific surface area. This allows us to justify why damage continues to occur after 60 min because, as mentioned above, a lower thickness and higher specific area facilitate fragmentation and damage generation during sonication. This fragmentation and damage facilitate the reaggregation of the C750 GNPs, which is the origin of the deterioration in dispersion as sonication time increases. Both effects lead to C750-containing nanocomposites losing orders of magnitude in electrical conductivity with sonication time.

### 3.3. Thermal Conductivity

The thermal conductivity of GNP/EP composites for all filler contents are presented in Figure S5 for 120 min of ultrasonication time. Initially, epoxy thermal conductivity was 0.20 W·m^−1^·K^−1^ at 25 °C. It is clearly observed that, for any GNPs loading, thermal conductivity is increased. Adding more filler improves thermal conductivity by increasing the number of filler particles in the polymer matrix and decreasing the distance between the filler particles. As has been suggested, thermal conductivity increases with fraction content of GNPs. The use of GNPs has been found to produce a higher increase on the thermal conductivity as compared with other carbon materials, such as CNTs [38]. This increase can be attributed to its two-dimensional structure, which can reduce the phonon scattering at the polymer–nanofiller interface [21].

**Figure 3:**
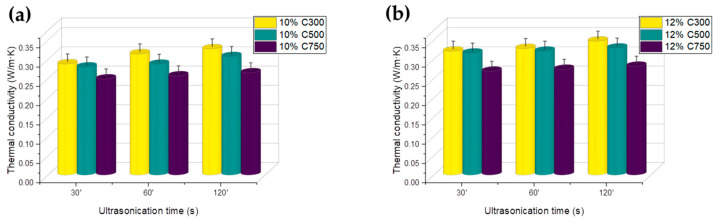
Thermal conductivity of GNP/epoxy composites as a function of loading and ultrasonic dispersion time for (**a**) 10% loading and (**b**) 12% loading.

Particularly for 10 and 12%, as shown in Figure 3, thermal conductivity reaches the highest values for C300, up to 0.34 W·m^−1^·K^−1^. A trend is observed in which the ultrasonication time increases thermal conductivity; this is because an increase in sonication time reduces the sedimentation size (Table S2). In this case, the thermal conductivity measurement is not taken in the xy direction, as was the case for electrical conductivity, but in the z direction, so that a higher sedimentation would imply a lower effective percentage of GNP dispersed in the matrix, thereby decreasing the thermal conductivity.

A second trend is observed in which the highest thermal conductivity corresponds to those with lower specific surfaces. This can be attributed to other parameters, such as the particle dispersion, which is better for C300, followed by C500 (Figure S10). In addition, the higher surface area of C750, which shows lower thermal conductivity, might be consistent with a stronger phonon scattering, causing a decrease in the thermal conductivity [21]. It may also be attributed to the fact that a smaller lateral size of the GNP usually leads to a lower thermal conductivity in the nanocomposites, so that the influence of lateral size would dominate over the specific surface area.

In addition, with the higher filler loading, a higher GNP content forms a continuous network, which is independent of the aspect ratio. In fact, it has been observed that the use of fillers with a high surface area shows a decrease in conductivity, both electrical and thermal. This is attributed to the increase in the resistance between the filler-to-filler connection points and the filler/polymer interfacial area. Shen et al. [39] employed molecular dynamic simulation and observed a decrease in thermal resistance at the graphene/epoxide interface with an increasing number of graphene layers, with C300 having the highest number of layers, followed by C500.

### 3.4. Morphology Results

It is well known that a nonuniform distribution of GNPs embedded into a polymeric matrix can negatively affect both mechanical and thermoelectric properties. GNPs, due to their relatively large size, can settle under the influence of gravity during the curing treatment before reaching the gelation state, therefore showing a gradient variant of nanoparticle concentration from top to bottom [40]. Thus, the dispersion quality of GNPs was assessed by means of SEM, by measuring the sedimentation layer thickness, as shown in Figure S6 for 10% and in Figure S7 for 12% GNP loading. For that purpose, the samples were prepared to reveal their cross-section. The panoramic (stitching) images allow us to observe the sedimentation of GNPs near the bottom surface. This region ranges within the micrometer size, and the specific values are shown in Table S2. A larger surface area apparently decreases the formation of the sedimentation layer. The justification is to be found in the correct interpretation of the object of Stokes’ law because it is not the individual particles that settle but the agglomerates that form them. As agglomerate formation is more intense for C750 (Figures S6, S7, and S10), Stokes’ law indicates that the settling velocity of these particles will increase with the square of their radius; the larger the agglomerates, the greater the radius. On the other hand, the influence of sonication is also clear when comparing Figures S6 and S7. The layering layer decreases up to 85% in size for a 120 min sonication time. Agglomerate formation and self-stratification decrease with increasing sonication time due to the fragmentation/breakage of the graphene nanoplatelet agglomerates.

It is important to realize the differences between 10% and 12%, as observed in Figures S6 and S7. With higher loading, the sedimentation layer increases; thus, selecting high concentrations of GNPs can increase the anisotropy of the composites, thereby affecting their properties. It also generates the existence of a dynamic percolation threshold, as the percentage of graphene generates changes in viscosity, agglomerate size, and sedimentation, which in turn, affects the value of the electrical conductivity, which may be the origin of the lower slope observed after the percolation threshold in the figures of percolation thresholds for electrical conductivity (Figure S4). In addition, it has been demonstrated that the main disadvantage of the type C compounds is the high decantation that they present compared to others, such as types M and H. This phenomenon, as will be observed in all the subsequent figures, causes the particles to accumulate on the bottom of the compound, causing stress accumulation and a decrease in the properties [41].

Field emission gun-scanning electron microscopy (FEG-SEM) provides useful information about the topography of the samples and can be used to determine the graphene lateral size as covered by the actual standard for characterization of the structure of graphene [42], which could provide valuable information to understand its electrical properties. This is important to determine if the connectivity between the graphene platelets is improved. In Figure S8, the surface obtained from an azimuthal perspective can be observed, in which the dispersion stage of GNPs inside the epoxy matrix can be observed. The calculations concerning the lateral size are depicted in Table S3 for 10%. To avoid performing SEM on the 12% samples, the Raman-derived GNP lateral size data were used to extrapolate the lateral size of the smaller agglomerates that would be observed using Raman (Table S4). This is possible because it has already been shown that a higher percentage increases viscosity, resulting in less damage and fragmentation and a larger agglomerate size.

Clearly, lower sonication time shows higher lateral size, which is decreased upon sonication time. What is actually being measured are the lateral sizes of the smaller agglomerates, which mainly influence the electrical conductivity. Table S3 shows how the lateral size stagnates after 60 min of sonication for graphene C300 and C500, while for C750, the lateral size continues to decrease from 60 to 120 min of sonication. This would seem to indicate that a larger specific area would imply a higher fragmentation capacity and that a smaller lateral size lengthens the damage effect over time or delays it due to the numerous points of stress accumulations linked to the wrinkles commonly present in this type of graphene (wrinkles are explained both from a chemical point of view, due to the greater presence of oxygen, and from a physical point of view, presenting a smaller lateral size and a larger surface area, and the existence of wrinkles is necessary). In addition, the damage and fragmentation are also prolonged in time (i.e., the damage per unit time is lower) due to the lower probability of a direct collision of the jet, delaying in time the reach of the damage and fragmentation stabilization zone observed at C300 and C500.

### 3.5. Optical Microscopy Characterization (TOM)

**Figure 4:**
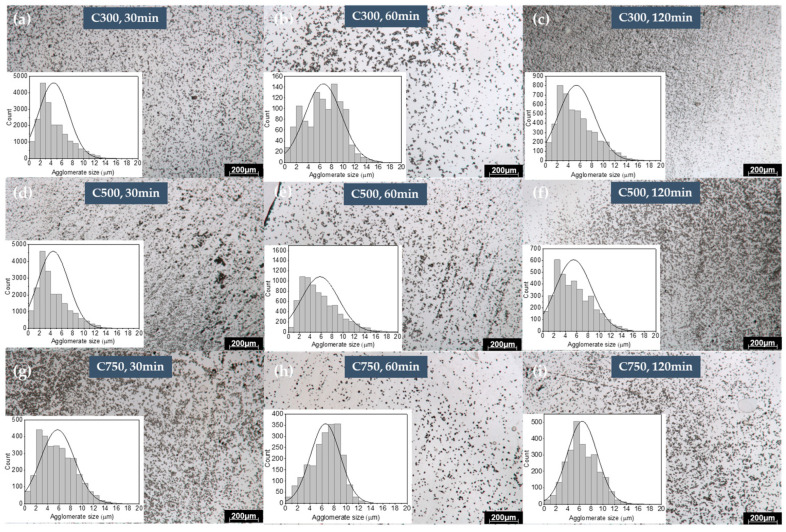
TOM images of samples with 12% loading and variable ultrasonication time (**a**–**c**) corresponds to C300 (30, 60, and 120 min), (**d**–**f**) corresponds to C500 (30, 60, and 120 min), and (**g**–**i**) corresponds to C750 (30, 60, and 120 min). Each image presents the histogram of the agglomerate sizes.

Transmission optical microscopy allows us to determine the mean agglomerate size of GNPs in the GNP/epoxy samples. Figure S9 shows the process employed to obtain the mean agglomerate size from the optical images. From Figure 4, it can be determined that, for C300 and C500, 60 min led to the higher agglomerate size, which decreases with further sonication time. C750 shows an increase in the mean agglomerate size with sonication time. As the sonication time increases, an increase in agglomerate size is observed due to the existence of a re-agglomeration stage caused by folds and creases in the graphene sheets, so that van der Waals interactions increase, helping to compensate for the bending energy in the folds by means of adhesion between sheets (re-agglomeration) [43,44]. A final stage of deagglomeration is observed at C300 and C500. It is also remarkable that the electrical conductivity shows a similar trend as the mean agglomeration size (Figure S10). This is observed for each graphene type, which demonstrates that the electrical conductivity is dominated by the formation of graphene pathways.

### 3.6. X-ray Diffraction and Raman Spectroscopy Characterization

**Figure 5:**
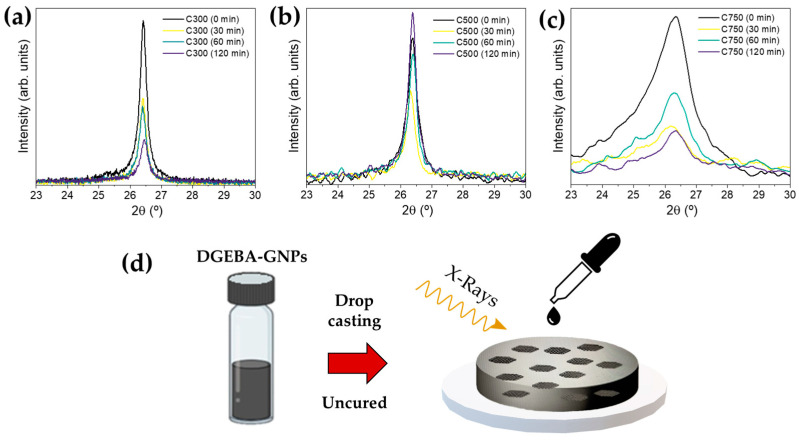
XRD diffractograms of the 10% GNP/epoxy composites (**a**) C300, (**b**) C500, and (**c**) C750 for different ultrasonication times (0, 30, 60, and 120 min); (**d**) shows the schematic representation of the XRD measure, showing the incident X-rays over the GNPs/epoxy.

XRD consists of a nondestructive technique to analyze the filler presence and the phase purity, and it can even provide useful information regarding the number of layers of GNPs [45]. XRD was obtained on the uncured sonication extracted samples (Figure 5d), where the sonication effects are more pronounced and the information obtained is not biased by subsequent processes. The XRD shows the information collected for angles greater than 20° due to the fact that there is a broad peak between 10° and 20°, which is often referred to as the amorphous halo of the epoxy group [8].

The diffractograms are depicted in Figure 5. A broad peak can be observed around 26.4° degrees, corresponding to (002) of the crystalline carbon 3R phase (JCPDS file 96–120-0019) [46]. Moreover, the diffraction plane (004) can also be observed with a low intensity (detailed region shown in Figure S11) and, in the case of C750, the (100) plane at 44°, which evidences that it presents a higher disordered structure [47]. In fact, C750 shows lower crystallinity, as the diffraction peak is broad and presents low intensity. This agrees with EDS results. C750 shows higher oxygen content; as the oxidation level increases, the peak of graphene becomes less intense (due to defects) [48].

The mean crystallite size (D) can be calculated by employing the full width at half-maximum (FWHM) of the XRD peak using the Debye–Scherrer equation [49]. The results are shown in Table S5, as well as the estimated number of layers obtained by the method explained in [50]. It can be clearly observed (Figure S12) that there is a decrease in the number of layers with sonication time (for instance, C300 decreases from 113 to 94 after 120 min of sonication). Table S6 shows the number of lamellae in the 12% nanocomposite, calculated from the Raman data of the I_2D_/I_G_ signal, which is directly linked to the exfoliation phenomenon. The trends as observed are maintained, and with increasing time, the number of lamellae decreases.

**Figure 6:**
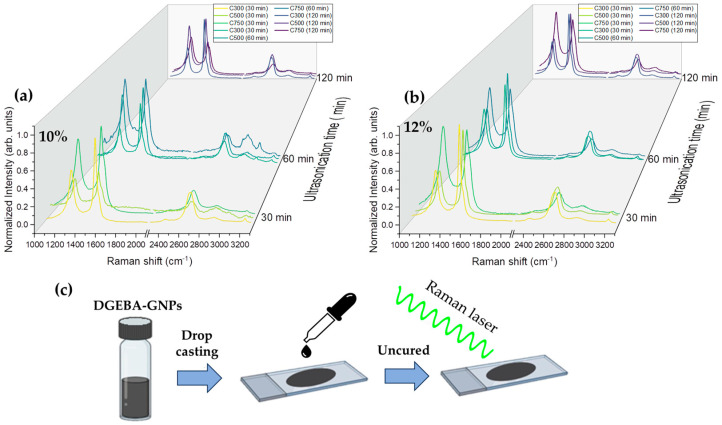
(**a**,**b**) Raman spectra of the GNP/epoxy composites as a function of the ultrasonication time for 10 and 12%, respectively. (**c**) Schematic representation of the Raman measure, showing the incident laser over the GNPs/epoxy deposited by drop casting.

Raman spectroscopy provides valuable information regarding the characterization of GNP/epoxy composites. Figure 6a,b shows Raman spectra of GNP/epoxy (10 and 12% loading) as a function of ultrasonication time. Raman bands corresponding to GNPs can be clearly observed with the appearance of a D-band (~1344 cm^−1^), G-band (~1582 cm^−1^), and 2-D (~2704 cm^−1^) band [45]. It should be mentioned that the D’-band appeared slightly as a shoulder merged with the G-band, both contributions being isolated by the use of Gaussian deconvolutions. Other modes related to the epoxy band can be observed also (~2925 cm^−1^) [51], and in 3065 cm^−1^.

The intensity ratio of the aforementioned peaks (*I_D_*/*I_G_*) constitutes a known quantitative index to determine the defect and fragmentation damage in carbonaceous materials such as CNTs and GNPs [52], often referred to as the “defect ratio”. This value is commonly expected, for graphitic materials with a low concentration of defects, to be around or lower than 0.2 [47]. By studying the D-to-G peak intensity ratio (*I_D_*/*I_G_*), as shown in Table S7, the lateral size (L_a_) in the graphene material can be estimated using the Tuinstra–Koenig relation [53] as follows:

(5) $$L_{a}(\mathrm{nm})=(2.4\cdot 10^{-10})\lambda_{L}^{4}{\left(\frac{I_{D}}{I_{G}}\right)}^{-1}$$

where $\lambda_{L}^{4}$ is the laser wavelength in nanometers and L_a_ is the average size of the crystallites. The estimated flake length (L_D_) can be obtained using [54] as follows:

(6) $$<L\geq 0.26{\left(\frac{I_{D}}{I_{G}}\right)}^{-1}$$

**Figure 7:**
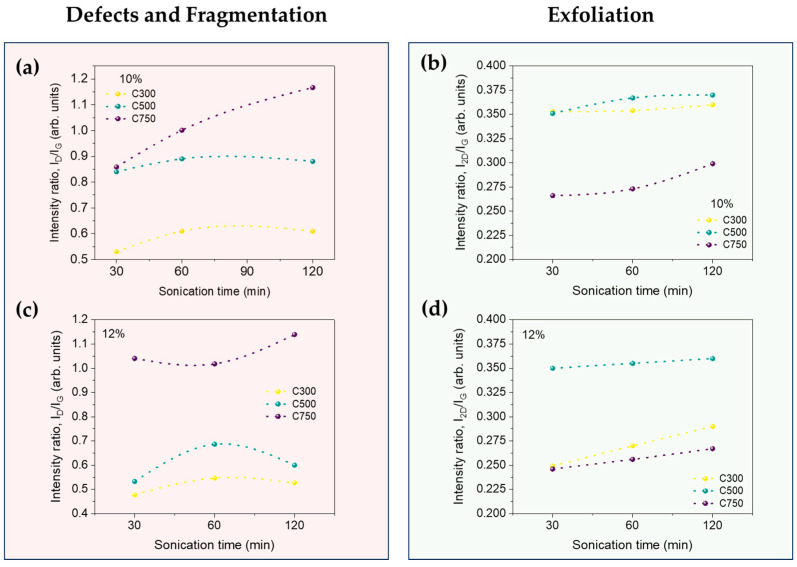
Evolution of damage and exfoliation with sonication time for the different GNPs at 10% and 12%. (**a**) Damage at 10%, (**b**) exfoliation at 10%, (**c**) damage at 12% and (**d**) exfoliation at 12%.

As observed in Figure 7, the defect ratio increases upon sonication time initially, from 30 min to 60 min. For instance, with 12% loading, C300 *I_D_*/*I_G_* increased from 0.478 to 0.547, implying that sonication induces damage to the graphene structure. However, with higher sonication time (120 min), it decreases. The relation between defect ratio and sonication time (or amplitude) is complex. Baig et al. [24] observed two different behaviors over time with sonication time, where initially, the increase in the defect ratio was small, and then it sharply increases. The small decrease, which is in some cases almost negligible, could be explained due to the decrease in the graphene thickness, which induces less structural disorder. It is also noticeable how a higher surface area shows a higher *I_D_*/*I_G_* ratio (e.g., for 12% loading, C750 shows at 30 min a value of 1.041, while C300 shows a value of only 0.478). This is caused due to the fabrication of GNPs with low numbers of layers (and higher specific surface). Usually, it is necessary to increase the exfoliation of graphite with physical or chemical methods, which also implies the damage to the aromatic network, increasing the presence of the defectivity of the graphene lattice (% of sp^2^) [23].

As *I_D_*/*I_G_* is the inverse proportional of the length of graphene flakes, L_D_, with a higher surface and ultrasonication time, the size of the flake length decreases (Table S7). This is due to GNP fragmentation occurring during sonication.

The 2D band is influenced by the number of graphene layers, and for this reason, it is linked to the exfoliation process. The appearance of the 2D band and the relation between the former D and the 2D mode (*I*_2*D*_/*I_G_*) can be attributed to the exfoliation of multiple layers of graphene, with their intensity related to the number of layers. In fact, as shown in Figure 7, *I*_2*D*_/*I_G_* continuously increases, sharply increasing for a 120 min sonication time, indicating that exfoliation during the whole sonication process is operating [20].

**Figure 8:**
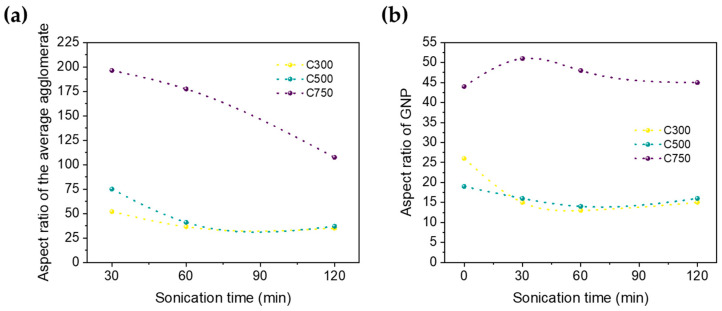
Evolution with sonication time of the aspect ratio of average (**a**) agglomerates and (**b**) GNP. The result is the average obtained from the 10 and 12% analysis.

With the aforementioned results, the aspect ratio (length/width ratio) of graphene and their agglomerates can be estimated (Figure 8). Lateral size has been obtained from SEM images (agglomerates) and Raman spectroscopy (GNP). While showing clear differences, the trends are very consistent; a higher sonication time induces a lower average lateral size, with lower thickness, which leads, in general, to a loss in aspect ratio. Only an increase in the aspect ratio is generated for graphene C750 after a sonication of 30 min, so that in general, for the rest of the cases, sonication has decreased the aspect ratio, and it can be concluded that sonication at times longer than 30 min is an excessively aggressive process for this type of graphene. Moreover, the larger the specific surface area of the graphene, the more suitable the sonication process is for dispersing it.

In all cases, higher sonication leads to a lower aspect ratio. For C300 and C500, the aspect ratio is very similar and thus is maintained. This is, however, not applicable to C750 because the aspect ratio does vary considerably and suffers a greater increase in damage with time. Similar behavior was observed for 12%, except that the damage in C750 is lower due to higher viscosity. As we already know, all electrical properties in nanocomposites are governed by percolation theories, which depend mainly on the aspect ratio. Therefore, the fact that GNP C750 shows a higher aspect ratio than C300 and C500 at first, justifies why it shows higher electrical conductivities and near-field electrical shielding. The same applies for C500, which shows a higher aspect ratio than C300.

### 3.7. Electromagnetic Shielding Interference

**Figure 9:**
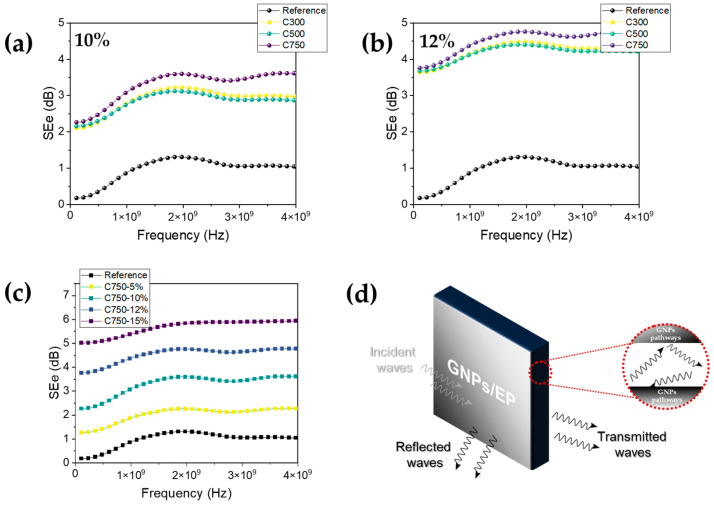
(**a**,**b**) EMI SE of the GNP/epoxy composites and their dependence with GNP type (C300, C500, and C750) for 10% and 12% loading, respectively (subindex “e” refers to electric field). (**c**) SE of the GNP/epoxy composites with C750 as a function of GNP loading. (**d**) Schematic representation of the EMI shielding phenomena showing the electromagnetic wave interaction with the GNPs/epoxy. All cases show 30 min sonication time.

Finally, the EMI shielding effectiveness of the different samples was analyzed in a near field configuration as a function of the sonication time and the GNP type in the range of 100 MHz to 4 GHz, as shown in Figure 9. Pristine epoxy shows an EMI shielding of around 1.0 dB maximum at near 2 GHz, showing minimum absorbance of electromagnetic (EM) waves. Figure 9a,b shows the influence of the surface area with two loadings, 10 and 12%. In any case, EMI shielding is increased with the increase in GNP content, especially for C750. This result can be attributed to the lower density and smaller lateral size of C750 graphene, which means that although the weight content is the same, the volume content is higher with respect to the C300 and C500 types, thereby increasing the number of C750 graphene in the matrix and therefore decreasing the probability of low graphene zones acting as easy transmission zones for EM waves. Thus, this causes the electromagnetic waves to be repeatedly absorbed or reflected, as well as being transferred into the conductive pathway [55]. It is important to understand the interaction between the EMI waves and the GNPs to understand the phenomena [56], in this case, is dominated by the presence of agglomeration, which have a larger size than the GNPs themselves. Both an increase in conductive networks with the addition of GNPs and the polarization-induced loss produces this effect. These losses increase not only with the GNP content but also with the surface area, as it has been recently reported [31]. A higher aspect ratio raises the percolation network (in this case, the larger the specific area, the higher the aspect ratio), which means that it has a higher electrical conductivity and thus increases the EMI shielding effectiveness [57]. It is important to note that this increase in shielding due to the increase in the electrical conductivity by the introduction of carbonaceous reinforcements can be preferably caused by reflection; however, the most desirable is to generate an adequate impedance mismatch to avoid these losses due to reflection and to increase the absorption losses, for example, by adding ferrites [13].

Although the results are not presented at higher sonication times due to lower shielding values, it is interesting to note that the absorbing character of these materials with higher sonication is greater than that of materials with lower sonication times due to the fact that the defects can favor the destruction of the charge distribution equilibrium, introducing a greater number of relaxations, increasing energy dissipation [58].

Figure 9c shows the influence of loading respective to SE for C750, showing the SEe for 0, 5, 10, 12, and 15%. With 15% loading, we obtained the maximum SE of 5.85 dB at 2 GHz. A representation of the phenomenon of shielding is depicted in Figure 9d. A portion of incident EM waves will be reflected from the surface of the composites. Meanwhile, the remaining EM waves enter the interior of the composites, where the presence of GNPs increases the multiple reflections of EM waves [56,59], where this energy is converted into electrical and thermal energy to dissipate the EM waves [60]. In our case, it would be the presence of agglomerates, which is greater in C750 and would originate from a greater contribution by multi-reflections and therefore would present a greater absorption of the signals in relation to C300 and C500. On the other hand, it has also been shown that multi-reflections are not the real cause of this increase in electromagnetic shielding in the GHz range but that the air contained in the polymer forms cells that increase the impedance match, so that the signal can penetrate and be absorbed [56].

Finally, a comparative table with the EMI shielding effectiveness reported in this work and the literature for similar systems can be found in Table S8 and in several reports, such as in [61].

## 4. Conclusions

Graphene-nanoplatelet–epoxy composites (GNP/EP) were fabricated through a simple method (gravity molding), and their properties (thermal and electrical) were studied, as well as their EMI shielding properties. Three different parameters have been studied, including graphene type (variable surface area, C300, C500, and C750), GNP loading (from 5 to 15%), and ultrasonication time (30, 60, and 120 min). TOM of uncured samples was used to study the quality of the dispersion, while the morphological changes induced in the nanoplatelets were studied using different techniques, including XRD, Raman, and FEG-SEM, allowing us to accurately measure their dimensions, lateral size and thickness (influenced by the layer number), and aspect ratio.

This study shows the complex nature of understanding the relation between the studied properties and morphology due to the demand and thoroughness of the characterization of the nanoplatelets and the interpretation of the results in order to correlate it. The dispersion by ultrasound modifies the morphological characteristics of the GNPs, and these in turn, affect the degree of dispersion on the polymer matrix. All these variables significantly influence the electrical percolation and EMI shielding behavior of these materials.

The research carried out in this work has laid the foundations for the effect of sonication on the dispersion and fabrication of GNP/polymer nanocomposites, which will be of great use to other authors in the design of this type of material, especially for electrical or magnetic applications.

The main specific conclusions drawn are as follows:

- A percolation threshold around 7.5% limits the use of lower contents for those applications, aiming this study at 10 and 12% loadings. Electrical conductivity is directly linked with the status of the dispersion of the GNPs into the EP matrix (the size of agglomerates) and with GNP properties (number of layers and lateral size).
- The sonication dispersion process affects the morphological characteristics of nanoplatelets; in addition to introducing mechanical defects, it reduces their lateral size and thickness through exfoliation, thereby modifying their aspect ratio.
- The relation between the defect ratio and sonication time is complex, depending on the initial morphological characteristics of GNP. A higher sonication time induces a lower average lateral size, with a lower surface area. This effect is a marker for GNPs with higher initial aspect ratios.
- Ultrasonication times higher than 30 min create low damage on GNPs, while higher sonication times produce a decrease in both the electrical conductivity and thus, the EMI shielding. This is related to the aspect ratio of graphene, which is higher for C750 and decreases with ultrasonication time.
- GNP/epoxy samples show an increase in EMI shielding in the range of 0.1–4 GHz, reaching a maximum of nearly 5 dB at 2 GHz. It has been confirmed that the efficiency of EMI shielding is directly proportional to the volume of nano-reinforcement added; therefore, it is favored by increasing the GNP content and increasing its aspect ratio.

Although, in the literature, it is common to use ultrasound as a method of dispersion of GNP to fabricate nanocomposites, it is important to know the influence of this physical process in the modification of their morphological characteristics because these then significantly affect the physical behavior of the final material.

## Figures and Tables

**Table 1:** Principal characteristics of GNPs employed in this work. Source: ^(a)^ Manufacture datasheet. ^(b)^ Ref. [26]. ^(c)^ Ref. [27].

GNP Type	Surface Area (m^2^/g) ^a^	Lateral Size (μm) ^a^	Thickness (nm) ^b^	Denisty (g/cm^3^) ^c^
GNP-300	300	1	4	2.33
GNP-500	500	0.5	2.8	2.30
GNP-750	750	0.3	1.9	2.21

**Table 2:** Principal properties of epoxy resin used in this work. All data have been extracted from the manufacture datasheet.

Epoxy Component	Viscosity at 25 °C (mPa s)	Density at 25 °C (g/cm^3^)	Epoxide Index (Eq/kg)	Amine Value (Eq/kg)
Araldite^®^ LY 556	11,000	1.175	5.375	-
Hardener XB 3473	102.5	1.05	-	11.65

## Data Availability

The data presented in this study are available on request from the corresponding author. The data are not publicly available due to privacy.

## Supplementary Materials

The following supporting information can be downloaded at: https://www.mdpi.com/article/10.3390/polym16081068/s1, Figure S1: TEM micrographs of the C300 GNPs; Figure S2: SEM micrographs of the C300 GNPs; Figure S3: STEM micrographs of the pristine GNPs (C300, C500, and C750); Figure S4: Percolation threshold in nanocomposites and 30 min of sonication; Figure S5: Thermal conductivity as a function of loading; Figure S6: SEM images of cross section of GNP/epoxy samples with 10% loading; Figure S7: SEM images of cross-section of GNP/epoxy samples with 12% loading; Figure S8: SEM images of samples with 10% loading and variable ultrasonication time; Figure S9: Diagram process to determine the agglomeration of GNPs within the GNP/epoxy composites by transmission optical microscopy (TOM); Figure S10: Mean average agglomerate size for each nanocomposite (C300, C500, and C750) for each ultrasonication time (30, 60, and 120 min); Figure S11: XRD diffractograms of the 10% GNP/epoxy composites; Figure S12: Evolution of the number of graphene sheets with sonication time for 10% GNP; Table S1. EDS analysis of each for the GNP; Table S2: Size of sedimentation layers (in µm; Table S3: Graphene lateral size obtained by SEM (in µm); Table S4: Results from the analysis of the crystallite size, lattice strain and number of layers obtained from XRD patterns for each of the 10% GNP/epoxy composites; Table S5: Results from the analysis of the crystallite size, lattice strain and number of layers obtained from XRD patterns for each of the 10% GNP/epoxy composites; Table S6: Equivalent graphene layer numbers obtained by extrapolation from the I_2D_/I_G_ obtained using Raman; Table S7: Results from the analysis of the Raman spectra. Table S8: Comparative table showing the EMI shielding effectiveness as compared with previous reports. References [62,63] are cited in the Supplementary Materials.
