# Supplementary material for: Ultrasonication Influence on the Morphological Characteristics of Graphene Nanoplatelet Nanocomposites and Their Electrical and Electromagnetic Interference Shielding Behavior
Source: Polymers (Basel). 2024 Apr 11;16(8):1068. doi: 10.3390/polym16081068 (PMC11054555; doi:10.3390/polym16081068)
Supplement: Supplementary file 1 [file polymers-16-01068-s001.zip › polymers-2952885-supplementary.pdf]

Supporting Information

Ultrasonication Influence on the Morphological Characteristics of Graphene Nanoplatelet Nanocomposites and Their Electrical and Electromagnetic Interference Shielding Behavior

Ignacio Collado [1,*], Alberto Jiménez-Suárez [1], Antonio Vázquez-López [1,*], Gilberto del Rosario [2] and Silvia G. Prolongo [1,3]

[1] Materials Science and Engineering Area, Escuela Superior de Ciencias Experimentales y Tecnología,
University Rey Juan Carlos, Tulipán Street, 28933 Móstoles, Madrid, Spain; alberto.jimenez.suarez@urjc.es (A.J.-S.); silvia.gonzalez@urjc.es (S.G.P.)

[2] Technological Support Center, University Rey Juan Carlos, Tulipán Street, 28933 Móstoles, Madrid, Spain; gilberto.delrosario@urjc.es

[3] Instituto de Tecnologías para la Sostenibilidad, Universidad Rey Juan Carlos, Tulipán Street,
28933 Móstoles, Madrid, Spain

* Correspondence: ignacio.collado@urjc.es (I.C.); antonio.vazquez@urjc.es (A.V.-L.)

Contents

Graphene Nanoplatelets (GNPs) characterization 2
Transmission Electron Microscopy (TEM) 2
Scanning Electron Microscopy (SEM) 2
Transmission-Scanning Electron Microscopy (STEM) 2
Energy Dispersion Spectroscopy (EDS) 3
GNPs/epoxy composites Characterization 3
Electrical conductivity 3
Thermal conductivity 4
Scanning Electron Microscopy 4
Graphene lateral size 6
Transmission optical microscopy (TOM) 7
X-Ray Diffraction (XRD) 9
Raman spectroscopy 11

Graphene Nanoplatelets (GNPs) characterization

Transmission Electron Microscopy (TEM)

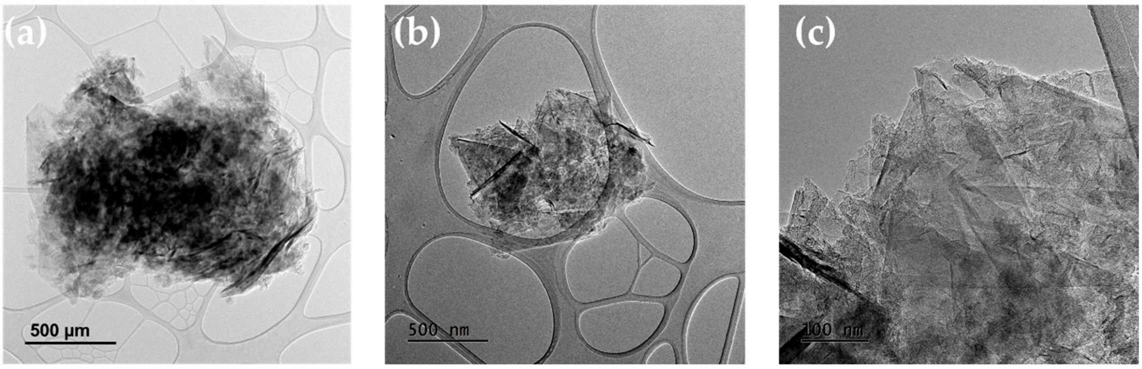

Figure S1: TEM micrographs of the GNPs (C300) (a) before and (b)-(c) after sonication 30 min. Note the scale difference.

Scanning Electron Microscopy (SEM)

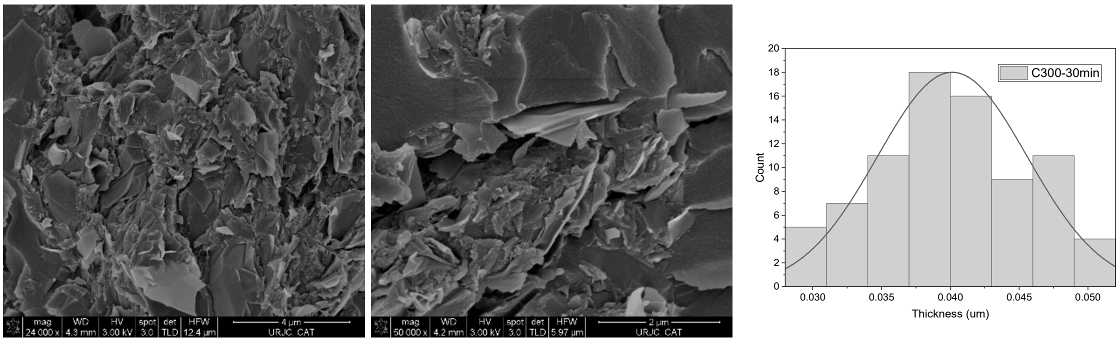

Figure S2: SEM micrographs of the GNPs (C300-30min) (a) 24000 x, (b) 50000 x and (c) thickness distribution.

Transmission-Scanning Electron Microscopy (STEM)

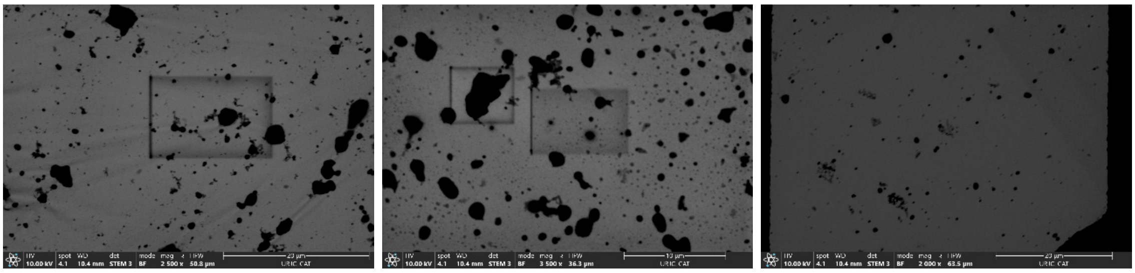

Figure S3: STEM micrographs of the pristine GNP (a) C300, (b) C500 and (c) C750.

Energy Dispersion Spectroscopy (EDS)

Table S1. EDS analysis of each for the GNP

Graphene Nanoplatelet	C (at.%)	O (at.%)	C/O ratio
C300	96.4	4.85	27.0
C500	95.1	6.6	19.2
C750	93.4	8.9	14.1

GNPs/epoxy composites Characterization

Electrical conductivity

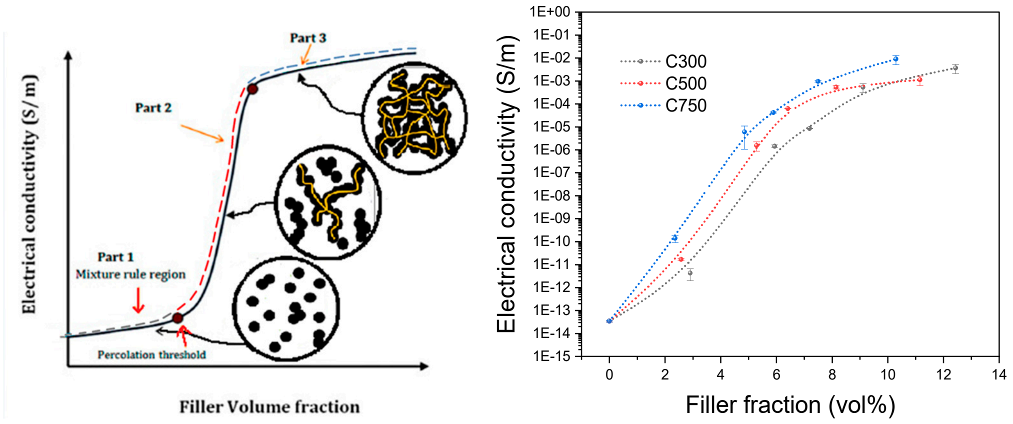

Figure S4. (a) Typical percolation threshold in nanocomposites and (b) Percolation threshold obtained for 30 minutes of sonication. The densities used for the calculation of the volume fraction are based on previous reports[1], being 2.33, 2.3 and 2.2 (g/cm^3) respectively.

Thermal conductivity

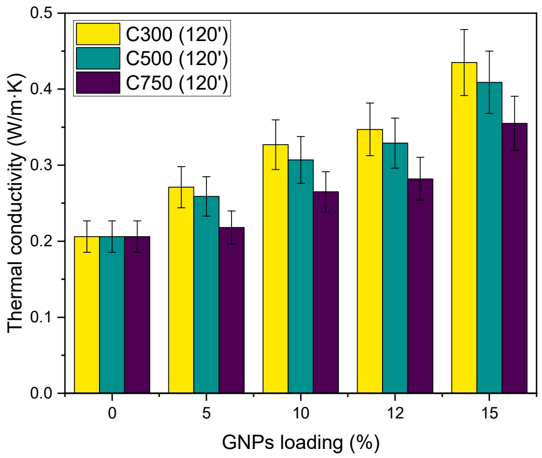

Figure S5: Thermal conductivity of GNPs/epoxy composites as a function of loading (from 0 to 15%). Ultrasonication time was set to 120 min in all cases.

Scanning Electron Microscopy

Table S2: Size of sedimentation layers (in μm).

**10% loading GNPs GNPs type/time**	**C300**	C500	C750
30 min	375.2±25.9	292.4±12.1	293.0±16.1
60 min	100.4±15.8	161.1±9.9	128.1±15.4
120 min	55.7±10.8	87.6±11.6	77.5±16.5
**12% loading GNPs GNPs type/time**			
30 min	362.8±9.5	365.1±10.1	456.8±14.6
60 min	240.9±18.7	240.8±16.8	237.6±22.9
120 min	201.9±18.1	157.4±4.5	129.0±24.7

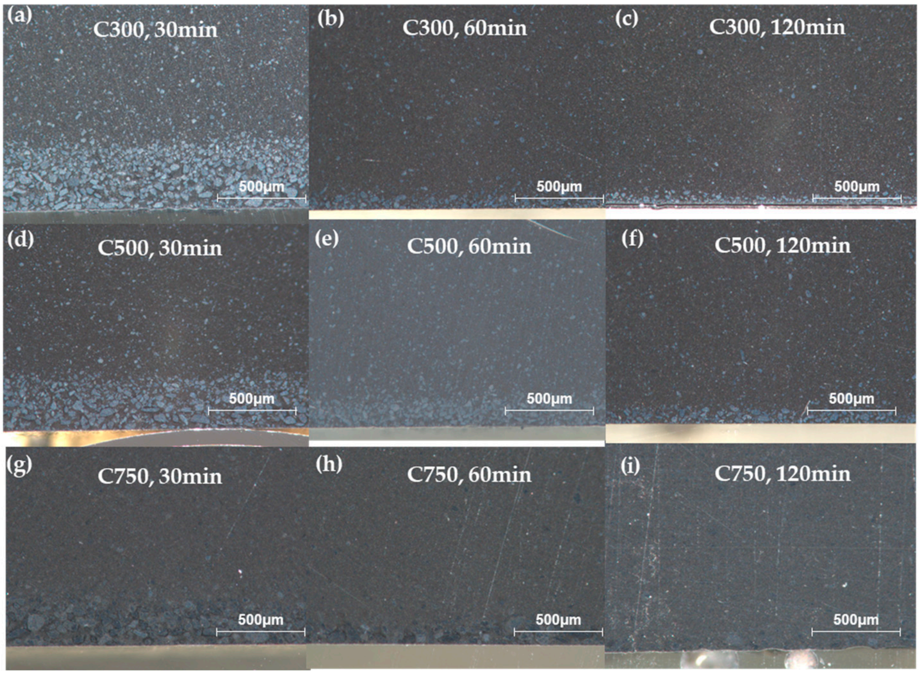

Figure S6. Colored panoramic SEM images of samples with 10% loading and variable ultrasonication time of the samples C300 (a), (b) and (c). (d),(e),(f) C500 and (g), (h), (i) to C750 corresponding to ultrasonication times 30, 60 and 120 respectively.

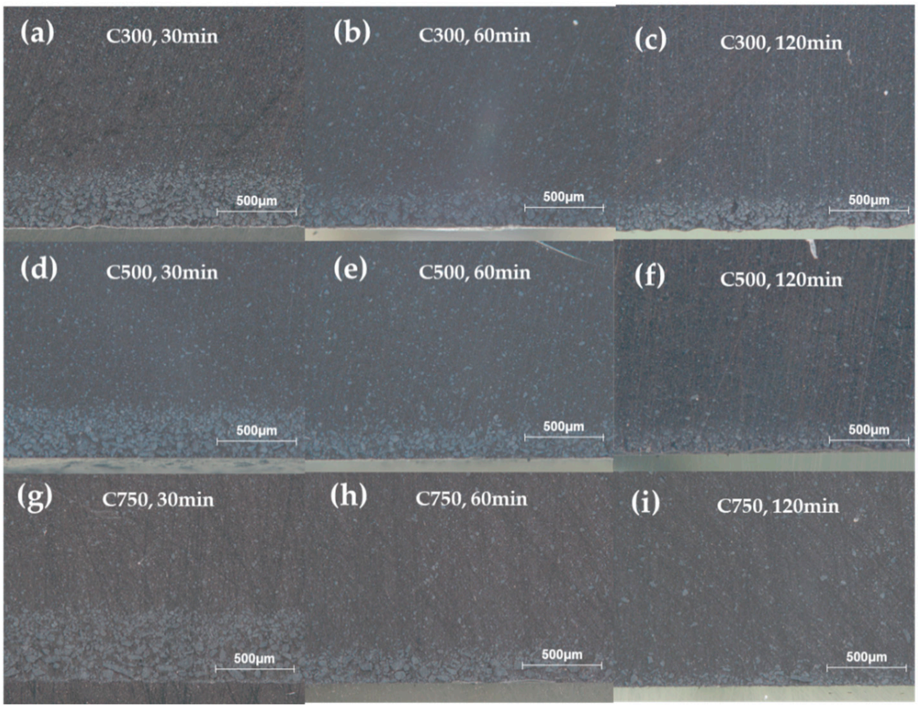

Figure S7: Colored panoramic SEM images of samples with 12% loading and variable ultrasonication time of the samples C300 (a), (b) and (c) corresponding to ultrasonication times 30, 60 and 120. (d),(e),(f) C500 and (g), (h), (i) to C750.

Graphene lateral size

Table S3: Graphene lateral size obtained by SEM (in μm).

**Interval size (um) (10%)**	**C300**						**C500**						**C750**					
	**30'**		**60'**		**120'**		**30'**		**60'**		**120'**		**30'**		**60'**		**120'**	
	**N**	**Fraction (%)**	**N**	**Fraction (%)**	**N**	**Fraction (%)**	**N**	**Fraction (%)**	**N**	**Fraction (%)**	**N**	**Fraction (%)**	**N**	**Fraction (%)**	**N**	**Fraction (%)**	**N**	**Fraction (%)**
0.25-0.5	0	0.00%	55	12.09%	75	19.08%	0	0.00%	40	29.20%	100	20.70%	20	7.35%	70	17.68%	100	74.63%
0.5-1	77	20.81%	231	50.77%	180	45.80%	75	63.56%	70	51.09%	235	48.65%	129	47.43%	200	50.51%	25	18.66%
1-2	188	50.81%	116	25.49%	105	26.72%	25	21.19%	20	14.60%	80	16.56%	86	31.62%	120	30.30%	9	6.72%
2-3	71	19.19%	38	8.35%	24	6.11%	10	8.47%	5	3.65%	3	0.62%	29	10.66%	6	1.52%	0	0.00%
3-4	25	6.76%	12	2.64%	7	1.78%	5	4.24%	1	0.73%	0	0.00%	8	2.94%	0	0.00%	0	0.00%
4-5	9	2.43%	3	0.66%	2	0.51%	3	2.54%	1	0.73%	0	0.00%	0	0.00%	0	0.00%	0	0.00%
Total	370	100%	455	100%	393	100%	118	100%	137	100%	483	87%	272	100%	396	100%	134	100%
**Average Lateral Size SEM (μm)**	**1.74**		**1.14**		**1.05**		**1.27**		**0.86**		**0.71**		**1.23**		**0.94**		**0.52**	

Table S4: Equivalent graphene lateral size obtained by obtained by extrapolation from the lateral size obtained by Raman.

	**C300**			**C500**			**C750**		
	30'	60'	120'	30'	60'	120'	30'	60'	120'
Lateral size Raman (10%) (μm)	0.490	0.423	0.429	0.309	0.297	0.285	0.303	0.260	0.223
Lateral size Raman (12%) (μm)	0.545	0.471	0.492	0.464	0.377	0.431	0.250	0.275	0.228
Proportional constant (12%/10%)	1.113	1.115	1.147	1.505	1.270	1.511	0.825	0.982	1.024

SEM lateral size (10%) (μm)	1.744	1.139	1.054	1.269	0.861	0.707	1.227	0.938	0.521
Equivalent SEM lateral size (12%) (μm)	1.940	1.271	1.209	1.909	1.094	1.068	1.012	0.820	0.533

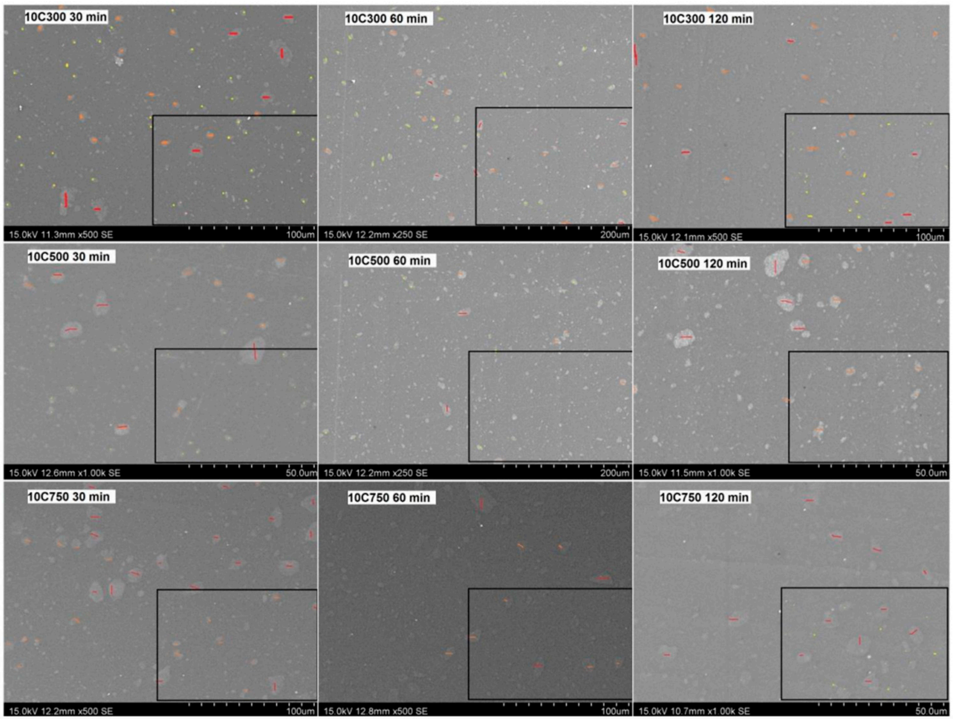

Figure S8. SEM images of samples with 10% loading and variable ultrasonication time.

Transmission optical microscopy (TOM)

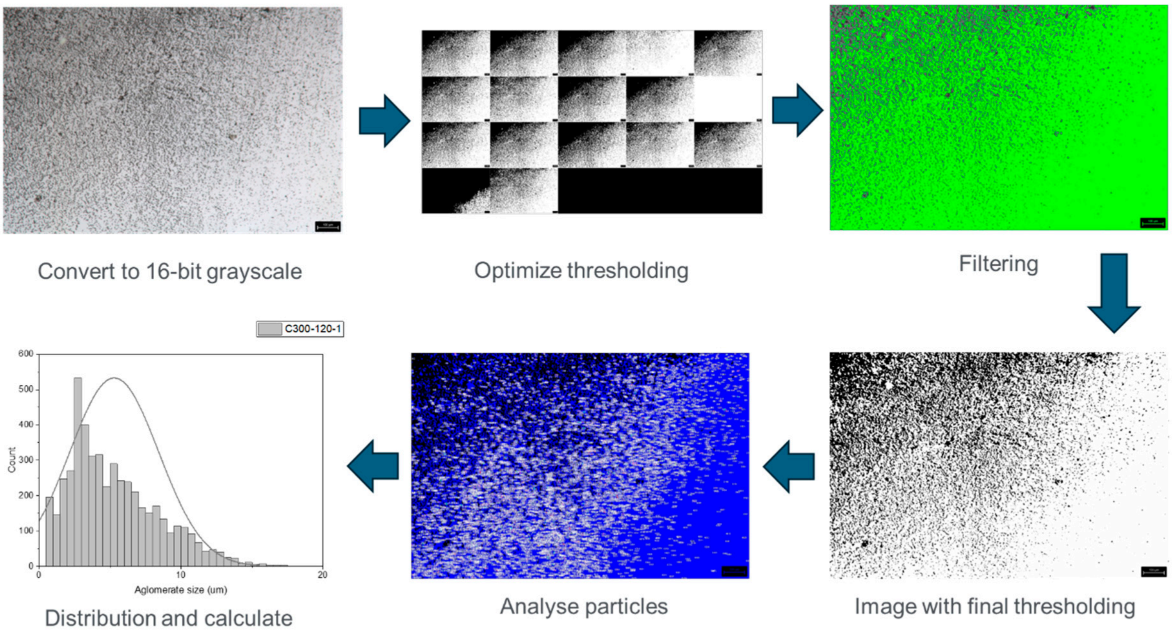

Figure S9: Diagram process to determine the agglomeration of GNPs within the GNPs/epoxy composites by Transmission Optical Microscopy (TOM).

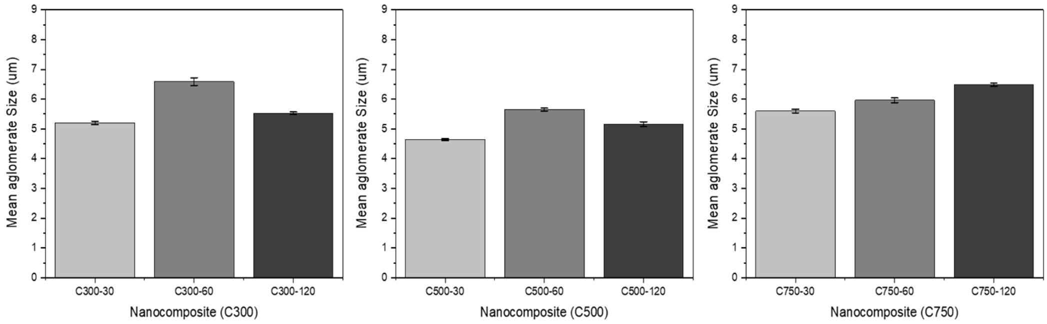

Figure S10: Mean average agglomerate size for each nanocomposite (C300, C500 and C750) for each ultrasonication time (30, 60 and 120 min)

X-Ray Diffraction (XRD)

Table S5: Results from the analysis of the crystallite size, lattice strain and number of layers obtained from XRD patterns for each of the 10% GNPs/epoxy composites.

		**2θ (°)**	**D (Å)**	**FWHM (°)**	**DScherrer (Å)**	**Lattice Strain (Å)**	**n (layers)**
**C300**	**0 min**	26.420	3.335	0.263	373.7	772.6	**113**
	**30 min**	26.400	3.335	0.282	343.3	709.6	**104**
	**60 min**	26.400	3.335	0.291	335.0	692.6	**101**
	**120 min**	26.450	3.335	0.310	320.4	662.8	**97**
**C500**	**0 min**	26.370	3.335	0.372	259.2	535.9	**78**
	**30 min**	26.338	3.335	0.379	251.6	520.1	**76**
	**60 min**	26.310	3.335	0.385	245.3	507.9	**74**
	**120 min**	26.365	3.335	0.399	241.3	499.4	**73**
**C750**	**0 min**	26.142	3.335	1.450	61.9	128.0	**20**
	**30 min**	26.010	3.335	1.683	51.6	106.6	**17**
	**60 min**	26.100	3.335	1.882	47.1	97.5	**15**
	**120 min**	26.240	3.335	2.118	43.6	90.2	**14**

Table S6: Equivalent graphene layer numbers obtained by extrapolation from the I_{2D}/I_G obtained by Raman.

	**C300**			**C500**			**C750**		
	30'	60'	120'	30'	60'	120'	30'	60'	120'
I_{2D}/I_G Raman (10%)	0.356	0.355	0.362	0.355	0.367	0.373	0.267	0.272	0.296
I_{2D}/I_G Raman (12%)	0.248	0.269	0.288	0.356	0.388	0.385	0.246	0.257	0.273
Propotional constant (10%/12%)	1.087	0.947	0.955	0.996	0.946	0.969	1.082	1.060	1.081
XRD graphene layer numbers (10%)	104	101	97	77	74	73	17	15	14
Equivalent graphene layer numbers (12%)	107	98	91	77	72	70	18	16	15

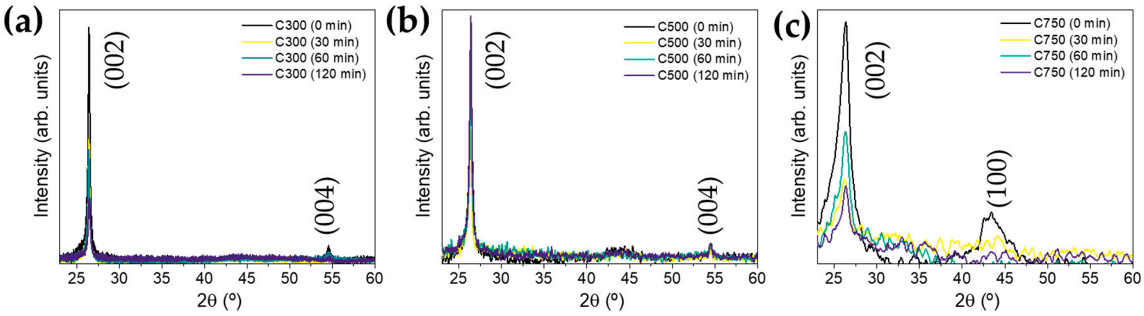

Figure S11: XRD diffractograms of the 10% GNPs/epoxy composites (a) C300 (b) C500 and (c) C750 for different ultrasonication times (0, 30, 60 and 120 min) in the range of 24-60°.

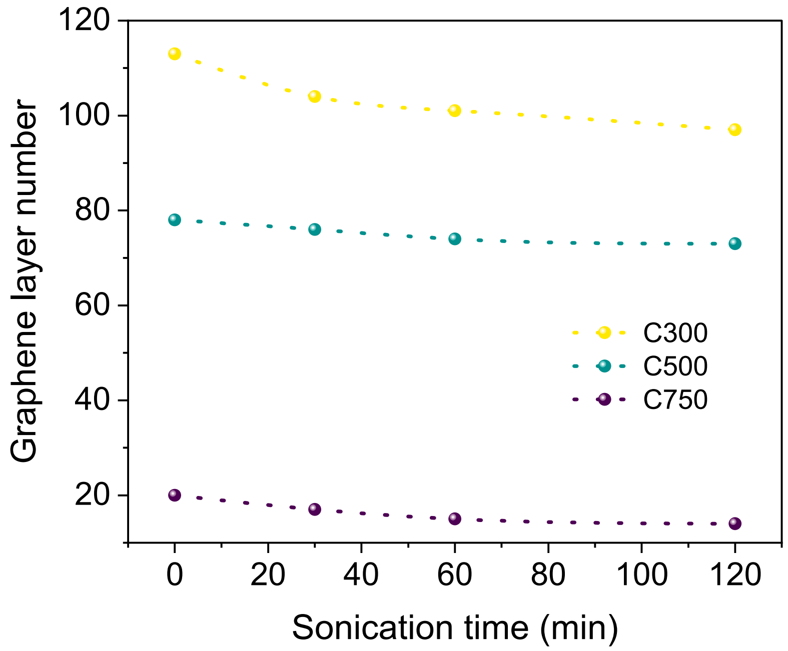

Figure S12: Evolution of the number of graphene sheets with sonication time for 10% GNPs loading for C300, C500 and C750 reinforced nanocomposites.

Raman spectroscopy

Table S7: Results from the analysis of the Raman spectra.

	**C300**			**C500**			**C750**		
**12% loading**	**I_D/I_G**	**I_{2D}/I_G**	**L (μm)**	**I_D/I_G**	**I_{2D}/I_G**	**L (μm)**	**I_D/I_G**	**I_{2D}/I_G**	**L (μm)**
30'	0.478± 0.023	0.249± 0.023	0.544± 0.021	0.533± 0.023	0.354± 0.023	0.487± 0.021	1.041± 0.023	0.247± 0.023	0.249± 0.021
60'	0.547± 0.023	0.268± 0.023	0.475± 0.021	0.687± 0.023	0.347± 0.023	0.378± 0.021	1.018± 0.023	0.257± 0.023	0.255± 0.021
120'	0.527± 0.023	0.288± 0.023	0.493± 0.021	0.600± 0.023	0.353± 0.023	0.433± 0.021	1.139± 0.023	0.263± 0.023	0.228± 0.021
**10% loading**	**I_D/I_G**	**I_{2D}/I_G**	**L (um)**	**I_D/I_G**	**I_{2D}/I_G**	**L (um)**	**I_D/I_G**	**I_{2D}/I_G**	**L (um)**
30'	0.526± 0.023	0.353± 0.023	0.494± 0.021	0.842± 0.023	0.351± 0.023	0.308± 0.021	0.857± 0.023	0.267± 0.023	0.303± 0.021
60'	0.611± 0.023	0.353± 0.023	0.426± 0.021	0.899± 0.023	0.367± 0.023	0.289± 0.021	1.001± 0.023	0.273± 0.023	0.259± 0.021
120'	0.594± 0.023	0.360± 0.023	0.438± 0.021	0.877± 0.023	0.373± 0.023	0.296± 0.021	1.168± 0.023	0.299± 0.023	0.222± 0.021

EMI Shielding

Table S8: Comparative table showing the EMI shielding effectiveness as compared with previous reports.

System	Reinforcement loading (wt %)	Thickness (mm)	EMI SE (dB)	Ref
Carbon black (CB)/epoxy Graphite /epoxy	7% graphite 7% CB	2	$SE_{(G)}$ of 17 dB $SE_{(CB)}$ of 21 dB	[2]
Single Walled Nanotubes/Epoxy	10 wt % SWNTs.	1.5	10-25 dB at 1GHz	[3]
Graphene/epoxy	10	0.1	4.5 dB at 11GHz 1 dB in the rest of GHz	[4]
Graphene and molybdenum disulfide nanosheets in Thermoplastic	0.3%	0.5	12 dB	[5]

polyurethane (TPU)				
GNPs/Epoxy	10 wt% GNPs	5	3.87 dB	[6]
GNPs/Epoxy	17 wt% GNPs	2	8 dB for 17% loading at 8 GHz	[7]
GNPs/Epoxy	15 wt% GNPs C750	1.8	5.85 dB at 2 GHz	This work

References

1. Dul, S.; Fambri, L.; Merlini, C.; Barra, G.M.O.; Bersani, M.; Vanzetti, L.; Pegoretti, A. Effect of Graphene Nanoplatelets Structure on the Properties of Acrylonitrile–Butadiene–Styrene Composites. *Polym Compos* **2019**, *40*, E285–E300, doi:10.1002/PC.24645.

2. Gümüş, E.; Yağımlı, M.; Arca, E. Investigation of the Dielectric Properties of Graphite and Carbon Black-Filled Composites as Electromagnetic Interference Shielding Coatings. *Applied Sciences 2023, Vol. 13, Page 8893* **2023**, *13*, 8893, doi:10.3390/APP13158893.

3. Li, N.; Huang, Y.; Du, F.; He, X.; Lin, X.; Gao, H.; Ma, Y.; Li, F.; Chen, Y.; Eklund, P.C. Electromagnetic Interference (EMI) Shielding of Single-Walled Carbon Nanotube Epoxy Composites. *Nano Lett* **2006**, *6*, 1141–1145, doi:10.1021/NL0602589.

4. Bontaș, M.G.; Diacon, A.; Călinescu, I.; Necolau, M.I.; Dinescu, A.; Toader, G.; Ginghină, R.; Vizitiu, A.M.; Velicu, V.; Palade, P.; et al. Epoxy Coatings Containing Modified Graphene for Electromagnetic Shielding. *Polymers 2022, Vol. 14, Page 2508* **2022**, *14*, 2508, doi:10.3390/POLYM14122508.

5. Khan, R.; Khan, Z.M.; Aqeel, H. Bin; Javed, S.; Shafqat, A.; Qazi, I.; Basit, M.A.; Jan, R. 2D Nanosheets and Composites for EMI Shielding Analysis. *Scientific Reports 2020 10:1* **2020**, *10*, 1–7, doi:10.1038/s41598-020-78614-6.

6. Dong, W.; Zhao, M.; Jin, F.L.; Park, S.J. Enhanced Electrical Conductivity and Electromagnetic Shielding Efficiency of Epoxy Resin Using Graphene Nanoplatelets. *Korean Journal of Chemical Engineering* **2022**, *39*, 1968–1974, doi:10.1007/S11814-021-1007-X.

7. Abdelal, N.; Dib, N.; Young, D.; Slanker, A. Electromagnetic Interference Shielding and Dielectric Properties of Graphene Nanoplatelets/Epoxy Composites in the x-Band Frequency Range. *J Mater Sci* **2022**, *57*, 13928–13944, doi:10.1007/S10853-022-07475-3/.
